# Molecular-Scale Plasmon Trapping via a Graphene-Hybridized Tip-Substrate System

**DOI:** 10.3390/ma15134627

**Published:** 2022-07-01

**Authors:** Guangqing Du, Yu Lu, Dayantha Lankanath, Xun Hou, Feng Chen

**Affiliations:** State Key Laboratory for Manufacturing System Engineering and Shaanxi Key Laboratory of Photonics Technology for Information, School of Electronic Science and Engineering, Xi’an Jiaotong University, Xi’an 710049, China; guangqingdu@mail.xjtu.edu.cn (G.D.); zjkly19900714@126.com (Y.L.); dayanthalankanath1@gmail.com (D.L.); houxun@mail.xjtu.edu.cn (X.H.)

**Keywords:** plasmon trapping, nanotip, plasmon potential well, high stability, graphene

## Abstract

We theoretically investigated the plasmon trapping stability of a molecular-scale Au sphere via designing Au nanotip antenna hybridized with a graphene sheet embedded Silica substrate. A hybrid plasmonic trapping model is self-consistently built, which considers the surface plasmon excitation in the graphene-hybridized tip-substrate system for supporting the scattering and gradient optical forces on the optical diffraction-limit broken nanoscale. It is revealed that the plasmon trapping properties, including plasmon optical force and potential well, can be unprecedentedly adjusted by applying a graphene sheet at proper Fermi energy with respect to the designed tip-substrate geometry. This shows that the plasmon potential well of 218 k_B_T at room temperature can be determinately achieved for trapping of a 10 nm Au sphere by optimizing the surface medium film layer of the designed graphene-hybridized Silica substrate. This is explained as the crucial role of graphene hybridization participating in plasmon enhancement for generating the highly localized electric field, in return augmenting the trapping force acting on the trapped sphere with a deepened potential well. This study can be helpful for designing the plasmon trapping of very small particles with new routes for molecular-scale applications for molecular-imaging, nano-sensing, and high-sensitive single-molecule spectroscopy, etc.

## 1. Introduction

In recent years, the plasmon trapping of nanoparticles with typical size of sub-100 nm less than light diffraction-limit has attracted great attention for both scientific and practical purposes. The plasmon trapping of molecular-scale nanoparticles can find a broad range of potential applications like molecular-imaging, nano-sensor, and single-molecule spectroscopy [1,2,3,4,5]. Physically, a special type of surface electromagnetic mode known as surface plasmon polariton can be excited from the origin of the surface electron resonance at designed geometrical nanostructures [6,7,8]. The surface plasmon polariton can be significantly squeezed into a very small size far beyond the optical diffraction limit, simultaneously bearing a large spatial gradient for supporting plasmon trapping. Consequently, a variation of geometry structures like nanotip, nanocavity, nano-bowtie, and nanopillar have been widely investigated for generating the localized surface plasmon modes for nanoscale particle trapping purposes [9,10,11,12,13]. Amongst them, the hybrid nanotip in particular exhibits great potential for flexibly controlling plasmon nanofocusing for nanoparticle trapping applications due to the intrinsic merit of a highly directional hotspot, as generated from the nanotip geometry [14,15,16,17,18].

To date, a series of investigations on the optical trapping in contact with the tip of probe have been carried out [19,20]. A theme considering substrate with a circular nanocavity is proposed to enhance the nanofocusing and optical trapping characteristics of the plasmonic tip [21]. Tip-functioned plasmonic nanocavity composed of the closely spaced silver coated fiber tip and gold film is proposed for producing radial vector mode, which can produce a nanosized near field with an electric-field intensity enhancement factor over 103 through exciting the plasmon gap mode in the nanocavity [22]. To improve the trapping ability, hybrid plasmon trapping has been actively investigated to squeeze light into a very small region for trapping a 20 nm-size sample [23]. The use of hybrid plasmonic trapping is motivated largely by its potential for considerably enhanced optical forces. This greatly improves well-functioning surface plasmon nanofocusing for trapping applications of nanotips. Despite the previous works, the tip-based trapping process is usually unstable due to the complex modulated scattering and gradient forces in proximity of a very small nanosphere with respect to possible apex misplacement in between tip-sphere system. Recently, graphene, a two-dimensional (2D) form of carbon in which the atoms are arranged in a honeycomb lattice, has been shown to possess unique properties with tunable surface plasmon excitations in a wide-range infrared wavelength regime. The plasmon resonance of graphene can be well manipulated by modifying the Fermi energy of the graphene for dramatically tuning the localized electric field [24,25,26,27]. It has the great advantage of manipulating electromagnetic gradient and scattering spectrum for plasmon trapping of small nanoparticles at nanoscale, potentially benefiting in terms of functioning nanoparticle trapping with possibly improved stability. So far, however, it still keeps an open topic for stable trapping of sub-50 nm particles via the graphene-hybrid nanotip due to the emergent Brownian motion significantly participating in the molecular-scale particles trapping process, deteriorating the plasmon trapping stability of nanotip system.

In this paper, we perform theoretical investigations on improving the stability for plasmon trapping of molecular-scale Au nanosphere with diameter of 10 nm by designing graphene-hybridized tip-substrate system. A hybrid plasmonic trapping model is self-consistently built for comprehensive predictions of plasmon trapping properties on the optical diffraction-limit broken nanoscale. It is proposed that the plasmon trapping potential around the targeted Au nanosphere can be unprecedentedly achieved to be as high as 218 k_B_T at room temperature via optimizing the surface medium film layer of the graphene-hybridized Silica substrate system. The results are well explained as the crucial role of Au-graphene hybridization participating in generating a highly localized electric field for supporting the high-stable trapping process. This study would be helpful for understanding the stable plasmon trapping properties for molecular-scale targets for advancing the potential applications in fields of molecular-imaging, nano-sensing, and single-molecule spectroscopy, etc.

## 2. Modeling and Methods

**Figure 1:**
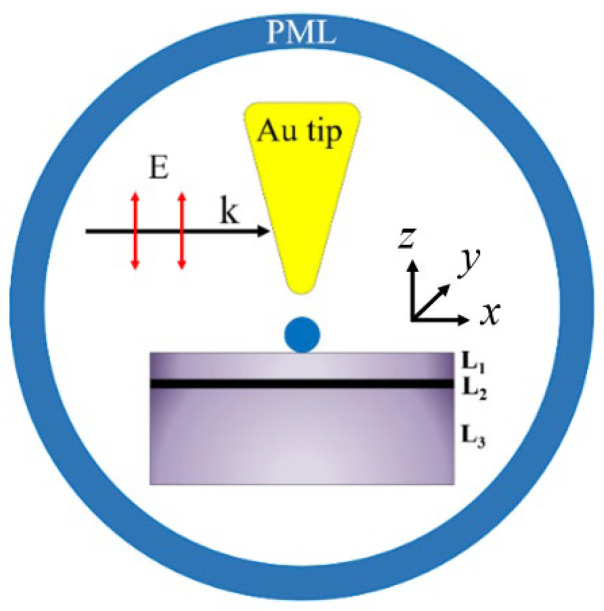
The schematic of 2-D hybrid plasmonic trapping model. L_1_ represents for surface medium film layer, L_2_ for graphene sheet, L_3_ for Silica substrate.

A 2-D hybrid plasmonic trapping model for the comprehensive predictions of plasmonic trapping properties is proposed, in which the nanotrapping geometry is made of an Au tip with a graphene sheet, which is embedded in between surface medium film and Silica substrate (see Figure 1). The Au sphere with diameter of 10 nm is put on the surface medium film in proximity of the Au nanotip. As a plane-wave laser with wave vector ‘k’ propagates parallelly along the surface of the substrate, the electric field of the laser will oscillate along the direction of the *z* axis. In this case, the confinement of local surface plasmon mode can exhibit radial symmetry distribution in local space around the *z* axis. So, the 2-D modeling is qualified for revealing the full 3D space properties of plasmon trapping potential well. The optical force originating from the gradient and scattering localized electric field can be physically generated from the hybrid plasmon system as laser wavelength matches to surface plasmon resonance frequency, which is mainly determined by the graphene-functioned tip-substrate configurations. On the other hand, the resonance wavelength of the plasmon can be closely affected by the graphene plasmon dispersion relation [28,29]. The plasmon trapping forces are calculated based on the dipole approximation, which can be determined as follows by considering a nanosphere of radius “a”: [30]

$$F=\frac{1}{4}\varepsilon_{0}Re\left\{\alpha_{0}\right\}\nabla {\left|E\right|}^{2}+\frac{n\sigma }{2c}\left\{E\times H^{*}\right\}+\frac{\sigma }{2}Re\left\{i\frac{\varepsilon_{0}}{k_{0}}\left(E\cdot \nabla \right)E^{*}\right\}$$

where $\varepsilon_{0}$ is vacuum dielectric permittivity, α_0_ is polarizability of a point-like particle, *E* is incident electric field, *n* is index of refraction for surrounding medium, σ is total cross-section of the particle, c the speed of light, *H* the incident magnetic field, *k_0_* the laser wave vector in free space. 

Here, we carefully consider the plasmonic optical forces exerting on Au sphere by building the self-consistent Helmholtz equation model integrated with optical forces calculations from three types of contribution of gradient forces, radiation pressure force, and polarization force. The gradient distribution of the localized electric field would play a crucial role in generating the plasmonic trapping force for the graphene-hybridized tip-substrate system. Namely, the gradient force facilitates spatial confinement in optical trapping dominance compared to the other two forces. Furthermore, the plasmon polarizability is a key factor that characterizes optical response due to the plasmonic interaction between the optical field and nanostructure that determines the strength of plasmonic optical trapping properties. The trapping potential resulting from the optical forces is a key factor that determines the stability of the optical trap, and it can be obtained by [31]

$$U\left(r_{0}\right)=\int_{\infty }^{r_{0}}F\left(r\right)\cdot dr$$

Numerically, we firstly build hybrid geometry of the tip-substrate system, in which three layers graphene sheets with thickness of 1 nm are embedded in the substrate. Here, the graphene with respect to the Fermi energy modifications are modeled with the surface optical conductivity considering the intra and inter band contributions [32,33]. Then the hybrid tip-substrate geometry, ambient medium is divided into many small meshes. The Helmholtz equation is discretized at every mesh point to form a large sparse matrix on the defined geometry. A perfect matching layer (PML) is set outside of the tip-substrate geometry. The scattered light from the tip-substrate is totally absorbed through the PML in the far field [Figure 1]. The boundary condition at the interface between the graphene sheet and Silica substrate is treated as a continuous one. We obtain the numerical solutions of the Helmholtz equation and optical force modeling via Commercial soft of COMSOL Multi-physics. Under the investigation, we will focus on the localized electric field enhancement behavior by introducing graphene sheet for plasmon trapping for 10 nm Au sphere via designing the graphene-hybridized Silica substrate for high-stability trapping purposes. In addition, this self-consistently modeling can possibly be enlarged for other 2D nanomaterials in addition graphene sheets, like honeycomb XBi and XBi3 sheets for advanced molecular scale plasmon trapping [34].

**Figure 2:**
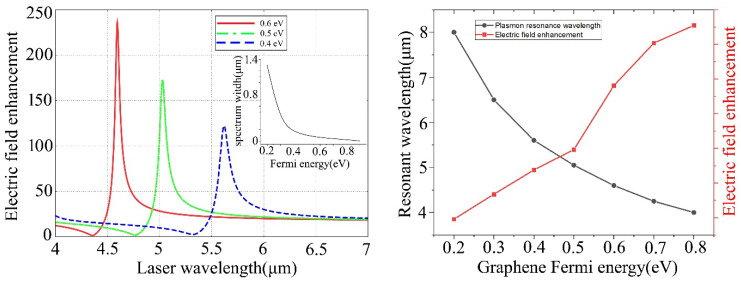
The localized electric field enhancement spectrum with respect to Fermi energy modification for graphene sheet of the graphene-hybridized tip-substrate system. The thickness of the surface Silica film layer is 10 nm.

Figure 2 shows the localized electric field enhancement spectrum with respect to the Fermi energy modification of a graphene sheet for the graphene-hybridized tip-substrate system. The graphene sheet is sandwiched in between the surface Silica film layer and Silica substrate. The trapping target is considered as a molecular-scale Au sphere with a diameter of 10 nm. It can be observed that, as a planar wave laser is horizontally incident on the tip-substrate system, the localized electric field enhancement defined as |E/E_0_| can achieve values as high as 240 at wavelength 4.65 μm by applying Fermi energy of 0.6 eV, as seen in Figure 2a. Also, the plasmon resonance wavelengths of the localized electric-field enhancement spectrum fall into the mid-infrared regime for the modified graphene Fermi energy of 0.4 eV, 0.5 eV and 0.6 eV, respectively. More interestingly, the electric-field enhancement spectrum profile width (Spectrum width) in the testing way of full width at half maximum (FWHM) becomes narrower by applying higher Fermi energy of the graphene sheet at 0.6 eV, as seen in the inset of Figure 2a, which is essentially important for high-sensitive wavelength-dependent sensor applications. We can see from Figure 2b that, as the applied Fermi energy of the graphene sheet increases from 0.2 eV to 0.8 eV, the localized electric field enhancement of |E/E_0_| can be remarkably further elevated for an even higher Fermi energy of 0.8 eV. Simultaneously, the plasmon resonance wavelength obtains an obvious blue-shift from 8 μm to 4 μm. The scale law of resonance wavelength vs. Graphene Fermi energy as $\lambda_{RW}\propto \;$*E_F_*^−1/2^ for a given wavevector q typically appears in the current result, originating from the intrinsic interband contributions to static graphene screening, which can be effectively absorbed in a background dielectric constant [28]. Also, the simulations show that the typical infrared resonance spectrum will totally disappear as the graphene sheets are removed. The electric-field enhancement spectrum with respect to modifying the graphene Fermi energy can be substantially helpful for a good understanding of the tunable-spectral properties of localized electric field enhancement exerting on the molecular-scale Au sphere target, which plays an important role in affecting the plasmon trapping properties on the diffraction-limit broken nanoscale for the graphene-hybridized tip-substrate system.

**Figure 3:**
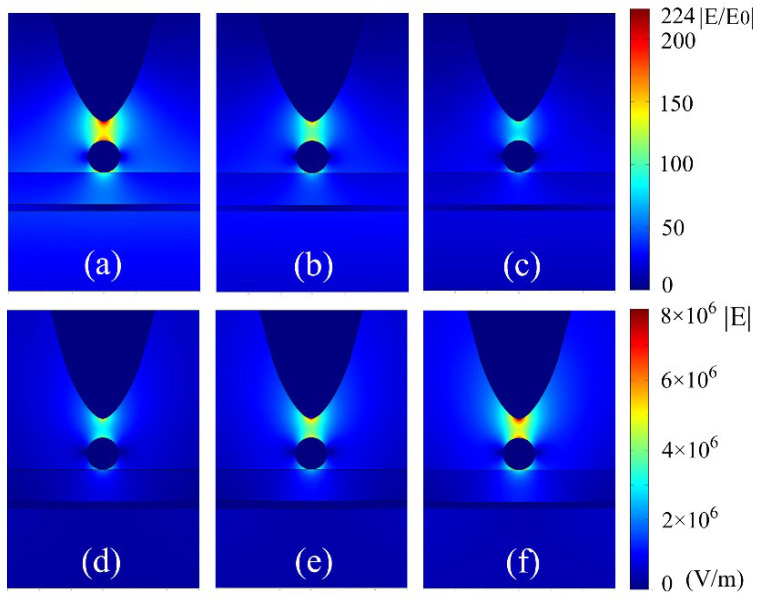
The 2-D images of localized electric-fields for the designed graphene-hybridized tip-substrate system with respect to Fermi energy of graphene sheet and external laser excitation. The Fermi energy is taken as 0.6 eV, 0.5 eV and 0.4 eV for (**a**–**c**), respectively. The Fermi energy is taken as 0.5 eV, and the electric field amplitude of external laser excitation is set as 3.5 × 10^4^ V/m, 4.5 × 10^4^ V/m and 5.5 × 10^4^ V/m for (**c**–**f**), respectively. The trapping Au sphere diameter is 10 nm, and the thickness of the surface Silica film layer is 15 nm.

The 2-D images of localized electric-fields for the designed graphene-hybridized tip-substrate system with respect to Fermi energy of graphene sheet and external laser excitation are shown in Figure 3. This clearly shows that the localized electric fields around the Au sphere with diameter of 10 nm are dominantly concentrated in the region of tip-sphere gap. In particular, we can clearly see that, at higher Fermi energy of 0.6 eV, the localized electric field is dramatically enhanced with large spatial gradient in proximity of a tip-sphere, as in Figure 3a. However, the localized electric field can be severely impaired across the tip-sphere gap as the Fermi energy of the graphene sheet is decreased from 0.45 eV to 0.3 eV, as seen from Figure 3b,c, respectively. This indicates that the spatial gradient element of the localized electric field becomes predominantly promoted for a higher Fermi energy of graphene sheet for the graphene-hybridized tip-substrate system. We can see from Figure 3d–f that the absolute value of localized electric field around the Au nanosphere can be further elevated to 8 × 10^6^ V/m at the applied electric field amplitude of 5.5 × 10^4^ V/m of the external laser excitation. Here, the external laser electric field amplitude is properly controlled to range from 3.5 × 10^4^ V/m to 5.5 × 10^4^ V/m in order to avoid laser local damage to the trapping system. It should be noticed that the localized electric field across the tip-sphere gap exhibits obvious large gradient distribution in cases of applying higher Fermi energy or larger electric field amplitude of external laser excitation. This indicates that the plasmon trapping process can be optimized by successfully designing the graphene-hybridized tip-substrate system, which can be very important for advancing a wide range of stable-controlled plasmon trapping applications.

**Figure 4:**
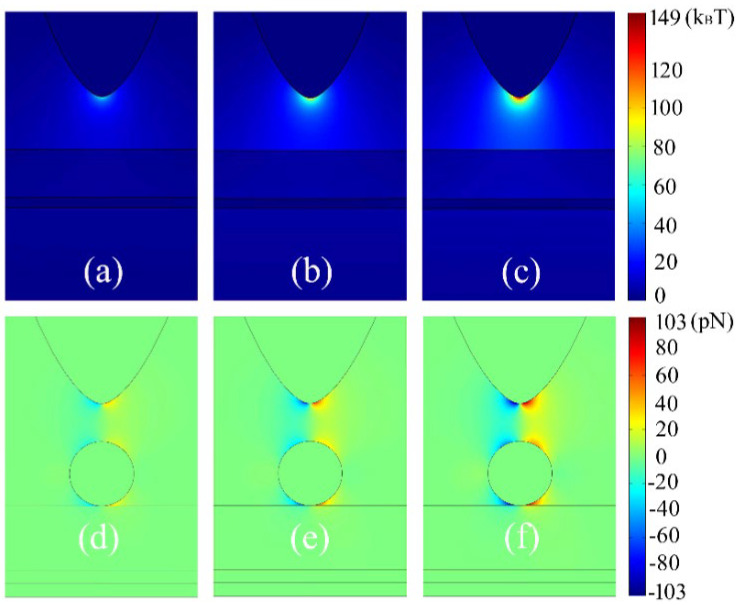
The plasmon trapping properties of potential well (**a**–**c**) and trapping force (**d**–**f**) with respect the electric field amplitude of external laser excitation. The trapping sphere diameter is 10 nm, the thickness of Silica film is 10 nm. The external laser excitation electric field amplitude is 3.5 × 10^4^ V/m for (**a**,**d**), 4.5 × 10^4^ V/m for (**b**,**e**), 5.5 × 10^4^ V/m for (**c**,**f**), respectively. The Fermi energy of graphene sheet is 0.6 eV, the thickness of the surface Silica film is 15 nm.

The 2D images of plasmon potential well for the graphene-hybridized tip-substrate system and the trapping force exerted on an Au sphere with respect to the electric field amplitude of external laser excitation are shown in Figure 4. We can see from (a)~(c) that the plasmon potential well across gap of the tip-substrate system can be significantly deepened as the laser electric field increases from 3.5 × 10^4^ V/m to 5.5 × 10^4^ V/m. The maximal potential well of 149 k_B_T can be achieved in proximity of tip vertex at the incident laser electric field of 5.5 × 10^5^ V/m. This is attributed to the strong plasmon hybridization of Au-graphene in the tip-substrate system assisted by reinforcement of augmented excitation of the external laser. Interestingly, as an Au sphere is put in the tip-substrate system, the quarter-poles distribution characteristics of plasmon force around the Au sphere can be observed, as seen from Figure 4d–f. In particular, the quarter-poles force around the Au sphere can be significantly enhanced by increasing the laser electric field from 3.5 × 10^4^ V/m to 5.5 × 10^4^ V/m. It should be emphasized that the trapping wavelength which is centered at 4.65 μm here has nothing to do with the external laser electric field. In fact, the quarter poles symmetry of trapping force together with the deepened plasmon potential well would play an active role in achieving the possible high stability of plasmon trapping for the potential applications of high-sensitive molecular-imaging, sensing, and SERS spectroscopy, etc.

**Figure 5:**
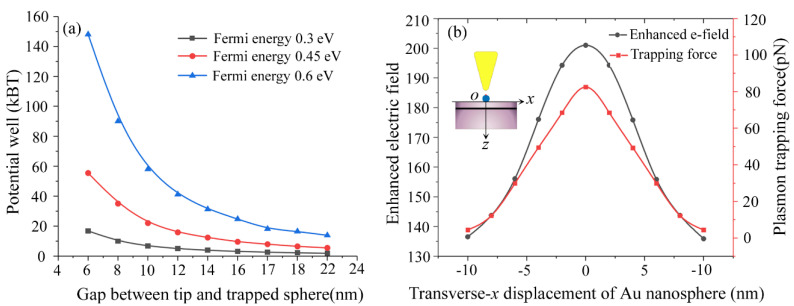
The plasmon trapping potential well and trapping force with respect to tip-to-sphere gap and the transverse displacement of Au nanosphere for the graphene-hybridized tip-substrate system. The trapping sphere is taken as 10 nm in diameter. We can see from (**a**) that the plasmon trapping potential extracting from vertex of the Au sphere drops rapidly as the tip-to-sphere gap increases from 6 nm to 14 nm for the graphene sheet Fer-mi energy of 0.6 eV. The plasmon trapping force as a function of the transverse displacement of Au nanosphere along surface of Silica substrate is shown in (**b**).

The plasmon trapping potential well and trapping force with respect to tip-to-sphere gap and the Au nanosphere transverse displacement at *x* direction for the graphene-hybridized tip-substrate system is shown in Figure 5. The Au sphere with diameter of 10 nm is considered as molecular-scale trapping target here. We can see from Figure 5a that the plasmon trapping potential extracting from vertex of the Au sphere drops rapidly as the tip-to-sphere gap increases from 6 nm to 14 nm for the graphene sheet Fermi energy of 0.6 eV. However, it shows a comparatively slower drop in the cases of the graphene sheet Fermi energy of 0.3 eV and 0.45 eV, respectively. Once the tip-to-sphere gap exceeds 14 nm, the plasmon trapping potential trends to achieve saturation with potential well less than 10 k_B_T for graphene sheet Fermi energy of 0.3 eV and 0.45 eV. Here, T is taken as 300 K in room temperature. This indicates that the plasmon trapping process becomes more and more unstable as the trapping potential falls into the saturation regime for the moderate graphene sheet Fermi energy of 0.3 eV and 0.45 eV, respectively. The result can be attributed to the unique role of graphene-functioned surface plasmon hybridization of the tip-substrate system, in which the surface plasmon resonance of the tip-substrate system can be significantly modified by varying the graphene sheet Fermi energy via a possible method of static electric doping [35]. The plasmon trapping force as a function of the transverse displacement of Au nanosphere along surface of Silica substrate is shown in Figure 5b. We can see that the plasmon trapping force exerted on the Au nanosphere with diameter of 10 nm can becomes as high as 80 pN at zero *x* displacement of the nanosphere from the tip apex. However, the plasmon trapping force can be badly impaired as the Au nanosphere displaces far from the tip apex. The localized electric field enhancement for supporting the plasmon trapping force exhibits a similar tendency with respect to the transverse displacement of Au nanosphere, which provides a basic understanding of the plasmon trapping force adjustment with respect to the localized electric field modifications for successfully designing and optimizing the displacement process of plasmon trapping for a molecular scale nanosphere with high stability.

**Figure 6:**
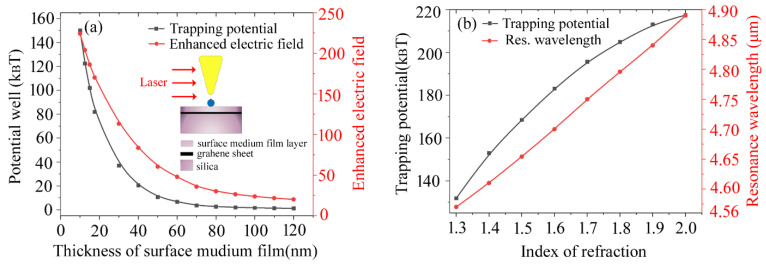
The plasmon trapping potential for the graphene-hybridized tip-substrate system with respect to the different surface medium film layers for the Silica substrate. (**a**) different thickness of surface medium film layer; (**b**) different index of refraction for the medium film layer. The trapping sphere with diameter of 10 nm is considered as the trapping target here. The applied Fermi energy for the graphene sheet is taken as 0.6 eV.

Figure 6 shows the plasmon trapping potential for the graphene-hybridized tip-substrate system with respect to modifications of the surface medium film layer, which is coated on a graphene-covered Silica substrate. Here, the plasmon potential well is extracted from the vertex of the trapping sphere for the graphene-hybridized tip-substrate system, as seen in the inset of Figure 6a. The plasmon trapping potential exhibits a rapid drop as the thickness of surface medium film layer is less than 60 nm. Nevertheless, it tends to achieve saturation once the thickness of the surface medium film layer exceeds 60 nm. This can be explained as the crucial role of graphene hybridization functioning in the Au-tip/Silica-substrate system, in which a thinner surface medium film layer can be substantially beneficial for boosting the hybridized surface plasmon interaction. The reinforcement of surface plasmon interaction leads to a significant electric field gradient for dominantly supporting the plasmon trapping force in together with deepening of the plasmon trapping potential as well. We can see from that Figure 6b that the plasmon trapping wavelength exhibits a near-linear rise as the index of refraction for the surface medium film layer increases from 1.3 to 2.0. More importantly, the plasmon trapping potential can be markedly amplified as increasing the index of refraction for the medium film layer. Generally, it is accepted that a stable trapping process can be expected as the plasmon trapping potential is far larger than k_B_T defined by the Brownian motion energy. Here, the maximal plasmon potential well can be elevated from 132 to 218 k_B_T via optimizing the index of refraction the surface medium film layer at a large value of 2.0 (see Figure 6b). The result indicates that extremely high trapping stability can be achieved via successfully optimizing the graphene-hybridized tip-substrate system, which would be helpful for advancing potential applications of single molecular-imaging, sensing, and single-molecule spectroscopy, etc.

## 3. Conclusions

We have theoretically investigated the achievable high-stability for plasmon trapping of Au nanosphere with a diameter of 10 nm by designing a graphene-hybridized Au-tip/Silica-substrate system. The localized electric field enhancement can achieve 240 by modifying the Fermi energy of the graphene sheet for the designed tip-substrate geometry. The maximal potential well of 149 k_B_T can be achieved in proximity of the tip vertex at the incident laser electric field of 5.5 × 10^5^ V/m. This is attributed to the strong plasmon hybridization of Au-graphene in the tip-substrate system assisted by the reinforcement of augmented excitation of the external laser. This shows that the plasmon potential well can be predominantly achieved in a range of 132 to 218 k_B_T by optimizing the index of refraction for the surface medium film layer on graphene covered Silica-substrate. This result can be critically important for understanding the high-stable plasmon trapping ability based on the graphene-functioned tip-substrate system for advancing the molecular-scale applications of nanoimaging, nanosensing, and SERS spectroscopy, etc.

## Data Availability

Not applicable.
